# Improving the visualization, interpretation and analysis of two-sample summary data Mendelian randomization via the Radial plot and Radial regression

**DOI:** 10.1093/ije/dyy101

**Published:** 2018-06-28

**Authors:** Jack Bowden, Wesley Spiller, Fabiola Del Greco M, Nuala Sheehan, John Thompson, Cosetta Minelli, George Davey Smith

**Affiliations:** 1 MRC Integrative Epidemiology Unit, Population Health Sciences, University of Bristol, Bristol, UK; 2 Institute for Biomedicine, Eurac Research, Bolzano, Italy; 3 Department of Health Sciences, University of Leicester, Leicester, UK; 4 Population Health and Occupational Disease, NHLI, Imperial College, London, UK

**Keywords:** Two sample summary data Mendelian randomization, Scatter plot, Heterogeneity statistics, Radial plot, Radial MR-Egger

## Abstract

**Background:**

Summary data furnishing a two-sample Mendelian randomization (MR) study are often visualized with the aid of a scatter plot, in which single-nucleotide polymorphism (SNP)–outcome associations are plotted against the SNP–exposure associations to provide an immediate picture of the causal-effect estimate for each individual variant. It is also convenient to overlay the standard inverse-variance weighted (IVW) estimate of causal effect as a fitted slope, to see whether an individual SNP provides evidence that supports, or conflicts with, the overall consensus. Unfortunately, the traditional scatter plot is not the most appropriate means to achieve this aim whenever SNP–outcome associations are estimated with varying degrees of precision and this is reflected in the analysis.

**Methods:**

We propose instead to use a small modification of the scatter plot—the Galbraith Radial plot—for the presentation of data and results from an MR study, which enjoys many advantages over the original method. On a practical level, it removes the need to recode the genetic data and enables a more straightforward detection of outliers and influential data points. Its use extends beyond the purely aesthetic, however, to suggest a more general modelling framework to operate within when conducting an MR study, including a new form of MR-Egger regression.

**Results:**

We illustrate the methods using data from a two-sample MR study to probe the causal effect of systolic blood pressure on coronary heart disease risk, allowing for the possible effects of pleiotropy. The Radial plot is shown to aid the detection of a single outlying variant that is responsible for large differences between IVW and MR-Egger regression estimates. Several additional plots are also proposed for informative data visualization.

**Conclusions:**

The Radial plot should be considered in place of the scatter plot for visualizing, analysing and interpreting data from a two-sample summary data MR study. Software is provided to help facilitate its use.

**Key Messages**

- Summary data furnishing a two-sample Mendelian randomization (MR) study are often visualized with the aid of a scatter plot. The scatter plot is also used to interpret the validity of the standard inverse-variance weighted (IVW) estimate and pleiotropy robust methods such as MR-Egger regression.
- A close relation of the scatter plot—the Radial plot—can instead be used for this purpose.
- The Radial plot removes the need to pre-process the summary data (a pre-requisite for MR-Egger), improves the detection of outliers and influential data points in either an IVW or MR-Egger analysis, and can incorporate any set of weights desired by the user.
- A more general form of MR-Egger regression is proposed that flows from, and naturally compliments, the Radial plot.
- Radial funnel and leave-one-out analysis plots can also be used to aid the visualization and interpretation of MR studies.

## Background

Mendelian randomization (MR)^1^ is a methodological framework for probing questions of causality in observational epidemiology using genetic data—typically in the form of single-nucleotide polymorphisms (SNPs)—to infer whether a modifiable risk factor truly influences a health outcome. A particular MR study design gaining in popularity combines publically available data on SNP–exposure and SNP–outcome associations from separate but homogeneous cohort studies of unrelated individuals for large numbers of uncorrelated SNPs. Each SNP is used to estimate the causal effect under the primary assumption that it is a valid instrumental variable (IV), by dividing its SNP–outcome association by its SNP–exposure association to yield the ratio estimate. Secondary modelling assumptions are also required in order for this estimate to be consistent. Ratio estimates are then combined into an overall estimate of causal effect using an inverse-variance weighted (IVW) fixed-effect meta-analysis. This is referred to as the IVW estimate and the general framework as two-sample summary data MR.^2^^,^^3^ For further details, see Box 1.

**Box 1: Standard two-sample summary data MR analysis**

**The IV assumptions:** The canonical approach to MR assumes that the group of SNPs are valid IVs for the purposes of inferring the causal effect of an exposure, *X*, on an outcome, *Y*. That is, they are: associated with *X* (IV1); not associated with any confounders of *X* and *Y* (IV2); and can only be associated with *Y* through *X* (IV3). The IV assumptions are represented by the solid lines in the causal diagram below for a SNP *Gj*, with unobserved confounding represented by *U*. Dotted lines represent dependencies between *G* and *U*, and *G* and *Y* that are prohibited by the IV assumptions. The causal effect of a unit increase in *X* on the outcome *Y*, denoted by *β*, is the quantity we are aiming to estimate.

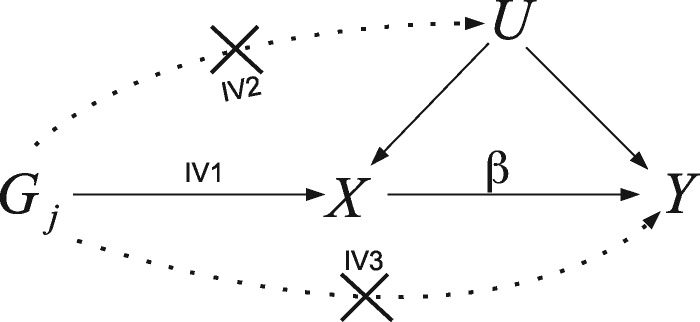

**The ratio estimate:** Assume that exposure *X* causally affects outcome *Y* linearly across all values of *X*, so that a hypothetical intervention that induced a 1-unit increase in *X* would induce a *β* increase in *Y*. Suppose also that all *L* SNPs predict the exposure via an additive linear model with no interactions. If SNP j is a valid IV, and the two study samples are homogeneous, then the underlying SNP–outcome association from sample 1, Γ_j_, should be a scalar multiple of the underlying SNP–exposure association estimate from sample 2, γ_j_, the scalar multiple being the causal effect *β*. That is:

$$\;\Gamma_{j}=\beta \gamma_{j}.$$

The ratio estimate for the causal effect of *X* on *Y* using SNP *j* (out of *L*), ${\hat{\beta }}_{j}$ = ${\hat{\Gamma }}_{j}/{\hat{\gamma }}_{j}$, where ${\hat{\Gamma }}_{j}$ is the estimate for SNP j’s association with the outcome (with standard error σ_Y*j*_) and ${\hat{\gamma }}_{j}$ is the estimate for SNP j’s association with the exposure (with standard error σ_Xj_).

**The IVW estimate:** The overall IVW estimate for the causal effect obtained across *L* uncorrelated SNPs is then given by

$${\hat{\beta }}_{IVW}=\frac{\sum_{j=1}^{L}w_{j}{\hat{\beta }}_{j}}{\sum_{j=1}^{L}w_{j}},$$

where *w_j_* is the inverse-variance of ${\hat{\beta }}_{j}$. Two popular choices for the inverse-variance weights are

$$1\text{st}\;\text{order}\;(\text{fixed}\;\text{effect})\;\text{weights}:\;\;w_{j}=\frac{{\hat{\gamma }}_{j}^{2}}{\sigma_{Yj}^{2}}$$

$$2\text{nd}\;\text{order}\;(\text{fixed}\;\text{effect})\;\text{weights}:\;\;w_{j}={\left(\frac{\sigma_{Yj}^{2}}{{\hat{\gamma }}_{j}^{2}}+\frac{{\hat{\Gamma }}_{j}^{2}\sigma_{Xj}^{2}}{{\hat{\gamma }}_{j}^{4}}\right)}^{-1}.$$

When SNP–exposure association estimates are sufficiently precise, so that $\sigma_{Xj}^{2}$ is negligible, or the causal effect *β* is small, then both weighting schemes are very similar. When this is not the case, both first- and second-order weights can perform poorly in terms of causal estimation and heterogeneity detection (see Box 2). ‘Iterative' and ‘exact’ weighting has recently been proposed by Bowden *et al.*^4^ to address this issue. For simplicity, first-order weights will be used throughout this paper. In our example, the IVW estimate obtained using first-order weights is 0.053. This represents the causal effect of a 1-mmHg increase in SBP on the log-odds ratio of CHD (see Table 1).

Different formulae for the inverse-variance weights can be employed, the most popular being simple ‘first-order’ weights, which assume the uncertainty in the SNP–exposure association estimates is negligible. Although more sophisticated weighting approaches have recently been proposed,^4^ for simplicity, we will use first-order weights throughout this paper.

### The scatter plot

**Figure 1.**
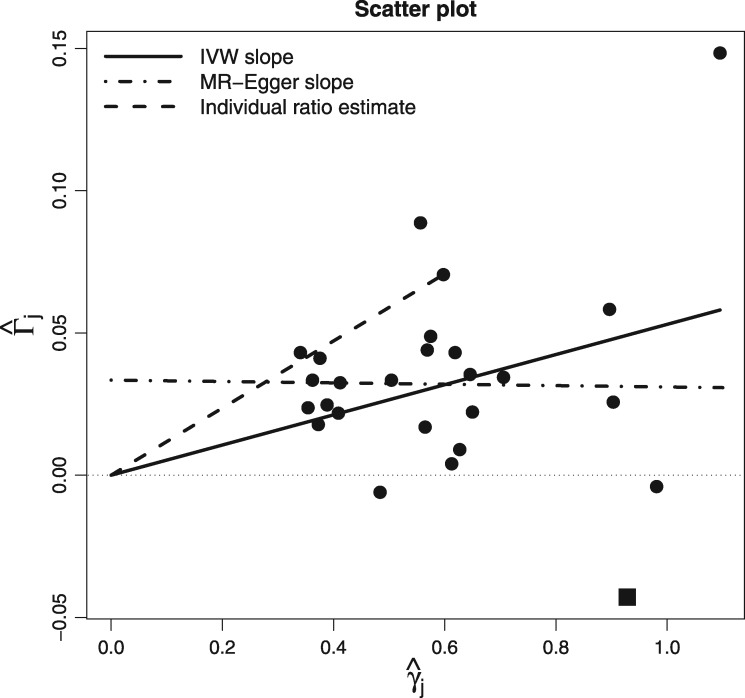
Traditional scatter plot of SNP–CHD associations ${\hat{\Gamma }}_{j}$ vs SNP–SBP associations ${\hat{\gamma }}_{j}$*.* SNP rs17249754 is shown as a square symbol.

Figure 1 shows a traditional scatter plot of summary data estimates for the associations of 26 genetic variants with systolic blood pressure (SBP, the exposure) and coronary heart disease (CHD, the outcome). SNP–SBP association estimates were obtained from the International Consortium for Blood Pressure consortium (ICBP).^5^ SNP–CHD association odds ratios were collected from Coronary ARtery Disease Genome-Wide Replication And Meta-Analysis (CARDIoGRAM) consortium^6^ and then transformed to the log-scale for subsequent model fitting. These data have previously been analysed and interpreted by Lawlor *et al.*^7^ and Bowden *et al*.^4^ They are included here for the purposes of illustration, rather than to draw any novel epidemiological conclusions.

The ratio estimate for any individual variant is the slope joining its data point to the origin, as shown for a single variant in Figure 1 (left). The IVW estimate for these data, which represents the causal effect of a 1-mmHg increase in SBP on the log-odds ratio of CHD, is 0.053. This is shown as the slope of a solid black line passing through the origin. The data point contributed by SNP rs17249754 is highlighted by a square symbol, as it will be subsequently discussed. It has become conventional to fix the sign of the SNP–exposure association estimates in these plots to be uniformly positive. This would naturally be achieved if each SNP had been coded to reflect the number of exposure-increasing alleles. SNP–outcome association estimates must also be checked and altered to account for this change (see Box 2 for further details). This does not alter the result of the IVW analysis, but makes it easier to interpret the IVW estimate as a best-fitting line through the data points.

**Box 2 Detecting and accounting for heterogeneity in two-sample summary data MR**

Heterogeneity amongst the ratio estimates can be calculated via Cochran’s *Q* statistic. When first-order weights are used for the *w_j_*, *Q* can be expressed in two ways:

$$Q=\sum_{j=1}^{L}Q_{j}=\sum_{j=1}^{L}\frac{1}{\sigma_{Yj}^{2}}{\left({\hat{\Gamma }}_{j}-{\hat{\beta }}_{IVW}{\hat{\gamma }}_{j}\right)}^{2}=\sum_{j=1}^{L}w_{j}{\left({\hat{\beta }}_{j}-{\hat{\beta }}_{IVW}\right)}^{2},$$

If heterogeneity is detected (*Q* much larger than *L*-1), this suggests violation of the modelling or IV assumptions. In our example, *Q *=* *67 and *L *=* *26, indicating substantial heterogeneity. Although horizontal pleiotropy is just one factor among many others that could be the underlying source of heterogeneity, we will assume it *is* the cause when explaining the implementation and assumptions of subsequent methods.

**Accounting for pleiotropy via a random-effects meta-analysis:** Let *α_j_* equal the pleiotropic effect of SNP *j* on the outcome *Y* not through *X*, with sample mean and variance across all *L* SNPs of $\mu_{\alpha }$ and $\sigma_{\alpha }^{2}$, respectively. If *α_j_* is independent in magnitude of the instrument strength across all SNPs (the InSIDE assumption) and $\mu_{\alpha }$= 0 (balanced pleiotropy), then an additive^24^ or multiplicative^25^ random-effects meta-analysis can be used to reliably estimate the causal effect and increase its standard error to reflect the additional uncertainty. In our analysis (Table 1), we take the multiplicative approach. This does not alter the IVW point estimate, but does increase its standard error by a factor of $\sqrt{\frac{Q}{L-1}}=\sqrt{67/25}\approx$ 1.64 relative to a fixed-effect model.

**Accounting for pleiotropy via MR-Egger regression:** If $\mu_{\alpha }$ is non-zero (directional pleiotropy), then the IVW estimate will generally yield a biased estimate for the causal effect. However, if the InSIDE assumption holds, then MR-Egger regression^11^ can still deliver reliable estimates for the causal effect, along with an estimate for $\mu_{\alpha }$. It is implemented by fitting the following linear regression of the SNP–outcome associations vs the SNP–exposure associations:

$${\hat{\Gamma }}_{j}=\beta_{0E}+\beta_{1E}{\hat{\gamma }}_{j}+\sigma_{Yj}\epsilon_{j},\;\;\text{where}\;\epsilon_{j}∼N(0,1)$$

after preprocessing the data according to the following rule:

$$\;\;\text{For}\;\text{all}\;j\;\text{in}\;(1,..,L)\;\text{such}\;\text{that}\;\;{\hat{\gamma }}_{j}<0:{\hat{\gamma }}_{j}\to -{\hat{\gamma }}_{j},\;{\hat{\Gamma }}_{j}\to -{\hat{\Gamma }}_{j}.$$

The standard implementation of MR-Egger regression tacitly assumes first-order weights. In this case, the InSIDE assumption is that the pleiotropic effects weighted by *σ_Yj_* are independent of the SNP–exposure associations weighted by σ*_Yj_*. In our example, the MR-Egger interval and slope estimates are 0.033 and –0.002, respectively (see Figure 1 and Table 1).

**Assessing heterogeneity about the MR-Egger fit:** Heterogeneity about the MR-Egger fit can be assessed using Rücker’s $Q^{\prime }$ statistic.^3^^,^^12^ When first-order weights are used for the *w_j_*, $Q^{\prime }$ can be expressed in two ways:

$$Q^{\prime }=\sum_{j=1}^{L}\frac{1}{\sigma_{Yj}^{2}}{\left\{{\hat{\Gamma }}_{j}-\left({\hat{\beta }}_{0E}+{\hat{\beta }}_{1E}\gamma_{j}\right)\right\}}^{2}=\sum_{j=1}^{L}w_{j}{\left({\hat{\beta }}_{j}-\frac{{\hat{\beta }}_{0E}}{\gamma_{j}}-{\hat{\beta }}_{1E}\right)}^{2},$$

Specifically, $Q^{\prime }$ tests for the presence of heterogeneity due to pleiotropy around the MR-Egger fit after adjustment for its mean value, $\mu_{\alpha }$ (estimated by ${\hat{\beta }}_{0E}$). This is equivalent to testing whether $\sigma_{\alpha }^{2}$ is greater than 0 (i.e. if the pleiotropic effects are not all identical). When such heterogeneity is detected, standard errors for the MR-Egger intercept and slope parameter estimates, ${\hat{\beta }}_{0E}$ and ${\hat{\beta }}_{1E}$, can be inflated by a factor of $\sqrt{1+{\hat{\sigma }}_{\alpha }^{2}}$. This is consistent with applying a multiplicative random-effects model using first-order weights. In our example, $Q^{\prime }$ = 58.6, indicating substantial heterogeneity (but less than for the IVW analysis). Standard errors are therefore inflated under a multiplicative model by a factor of $\sqrt{\frac{Q^{\prime }}{L-2}}=\sqrt{58.6/24}\approx$ 1.56 relative to a fixed-effect MR-Egger model.

### Detecting and adjusting for heterogeneity in two-sample MR

Within the meta-analytical framework underpinning the standard IVW estimate, heterogeneity observed amongst the ratio estimates can be assessed via Cochran’s *Q* statistic. If the necessary modelling assumptions hold for two-sample summary data MR and all SNPs are valid IVs, then Cochran’s *Q* should follow, asymptotically, a Chi-squared distribution, with degrees of freedom (df) equal to the number of SNPs minus 1. Excessive heterogeneity therefore points to a meaningful violation of at least one of these assumptions. Much attention has focused on detecting and adjusting for one specific source of violation referred to as *horizontal pleiotropy.*^8^^,^^9^ This occurs when SNPs exert a direct effect on the outcome through pathways other than the exposure. For brevity, we will refer to horizontal pleiotropy simply as ‘pleiotropy’ from now on.

Del Greco *et al*.^10^ first proposed the use of Cochran’s *Q* to detect pleiotropy in a MR context. However, the presence of heterogeneity due to pleiotropy does not automatically invalidate the IVW estimate. For example, if, across all variants:

- its magnitude is independent of instrument strength (the so-called ‘InSIDE’ assumption^11^);
- it has a zero mean (i.e. it is ‘balanced’);

then a random-effects meta-analysis can be used in lieu of the standard fixed-effects IVW meta-analysis to reliably estimate the causal effect accounting for the additional uncertainty due to pleiotropy. If (i) holds but not (ii), then MR-Egger regression can instead be used to reliably estimate the mean directional pleiotropic effect and causal effect.^3^^,^^11^ For the blood-pressure data in Figure 1, and assuming pleiotropy as the source of heterogeneity, MR-Egger regression estimates the mean pleiotropic effect (i.e. the intercept) to be 0.033 and the causal effect adjusted for pleiotropy (i.e. the slope) to be virtually zero. Thus, MR-Egger infers that the effect detected by the IVW approach is spurious and due to bias rather than any underlying causal mechanism.

An extended version of Cochran’s *Q* statistic (Rücker’s $Q^{\prime }$^3^^,^^12^) can be used to assess heterogeneity about the MR-Egger fit. See Box 2 for further details. The size of *Q* and $Q^{\prime }$ in relation to one another (specifically the difference $Q-Q^{\prime }$) gives an indication as to the relative goodness of fit of the IVW and MR-Egger models. For this reason, Bowden *et al.*^3^ suggest reporting the statistic *Q_R_* = $Q^{\prime }/Q$ to aid the interpretation of study results from an MR analysis. A *Q_R_* close to 1 indicates the IVW and MR-Egger models fit the data equally well, whereas a *Q_R_* much less than 1 indicates MR-Egger is best-fitting. They also adapt the hierarchical model-selection framework outlined by Rücker *et al.*^12^ for guiding which approach is appropriate for a given analysis. See Box 3 for further details. In essence, this framework favours the use of the IVW model over MR-Egger regression a priori because it yields causal estimates with higher precision, but recommends MR-Egger regression only when it provides a demonstratively better fit to the data.

**Box 3: The Rücker model-selection framework**

The Rücker model-selection framework^3^^,^^12^ is encapsulated in the diagram below.

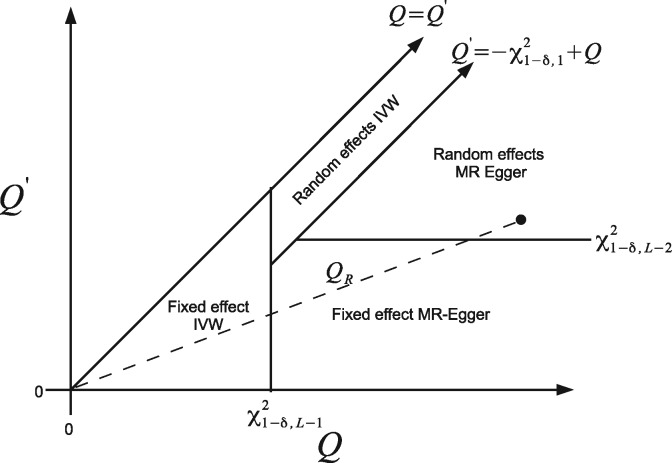

It shows the 2D decision space defined by *Q*, $Q^{\prime }$ and a significance threshold for detecting pleiotropy, δ (e.g. *δ* = 0.05). The rationale for this framework is briefly summarized:

1. Start by performing an IVW analysis under a fixed-effect model and calculate *Q*.
2. If *Q* reveals sufficient heterogeneity at significance level *δ* with respect to a $\chi_{L-1}^{2}$ distribution, then switch instead to a random-effects IVW model.
3. Fit fixed-effect MR-Egger regression and calculate $Q^{\prime }$. If the difference $Q-Q^{\prime }$ is significant at level *δ* with respect to a $\chi_{1}^{2}$ distribution, switch to this model.
4. If $Q^{\prime }$ reveals sufficient heterogeneity at significance level *δ* with respect to a $\chi_{L-2}^{2}$ distribution, then switch instead to a random-effects MR-Egger model.

For a given data set, the slope joining the point $\left(Q,Q^{\prime }\right)$ to the origin gives the ratio statistic *Q_R_* and the point $\left(Q,Q^{\prime }\right)$ immediately defines the selected model under the above framework. This is illustrated by the black dot in the diagram above. In this hypothetical case, the Rücker framework suggests the random-effects MR-Egger model is most appropriate.^3^ For the full data, random-effects MR-Egger regression is also suggested as the most appropriate method because *Q* = 67, $Q-Q^{\prime }$= 8.4 and $Q^{\prime }$ = 58 are all large compared with their respective null distributions. *Q_R_* is equal to 0.86. If a genetic variant is deemed to be sufficiently outlying to warrant removal from the MR analysis (e.g. like SNP rs17249754 in our example) the Rucker model selection framework must be repeated from the start.

Aligning the SNP–exposure association estimates to be positive is purely cosmetic for the IVW analysis, since the IVW estimate remains constant whichever coding is used. However, it is actually a necessary step for the standard implementation of MR-Egger regression. This can be understood by viewing MR-Egger as a method for detecting and adjusting for any systematic trend in the causal estimates according to the ‘weight’ each one receives in the IVW analysis, with weight being a strictly positive quantity.

### Limitations of the scatter plot for MR analysis

Although it has become the standard tool for visualizing summary data in an MR analysis, the scatter plot has a major limitation, which lies at the heart of this paper:

**The scatter plot does not give the most transparent representation as to the weight each genetic variant receives in the MR analysis, whenever the weights are not solely determined by the SNP–exposure associations**.

**Figure 2.**
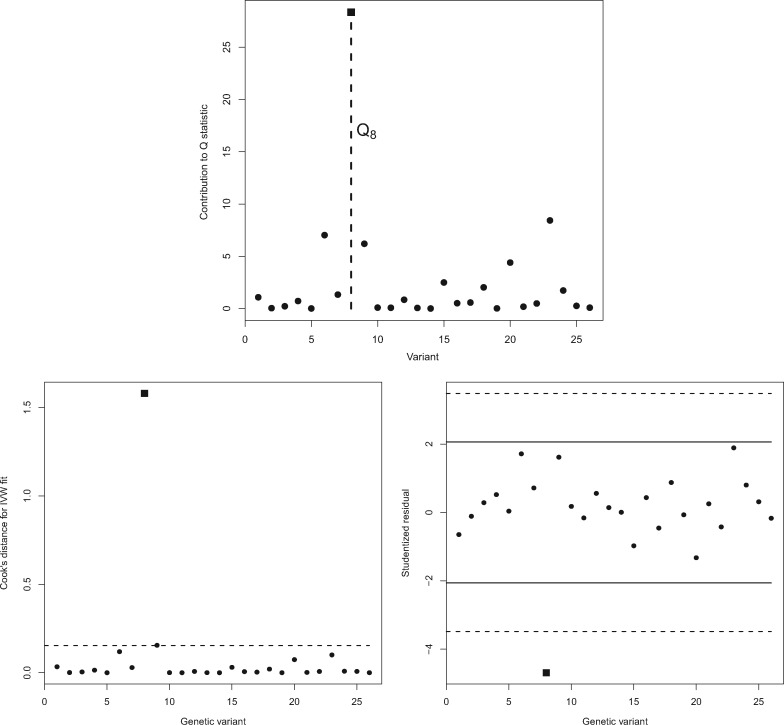
Top: Individual variant contributions to Cochran’s heterogeneity statistic. The contribution of SNP rs17249754 (labelled *Q*_8_) is shown as a square. Bottom left: Cook’s distance for each genetic variant in the SBP–CHD data, with standard influence threshold (4/#SNPs) indicated by a dashed line. Bottom right: Studentized residuals for each variant in the SBP–CHD data with standard 5% significance thresholds (solid black lines) and Bonferroni-corrected significance thresholds (5%/#SNPs, dashed lines). SNP rs17249754 is again shown as a square.

This is the case even when the IVW estimate is calculated using simple first-order weights, since they depend additionally on the SNP–outcome association standard error. The fitted slope in Figure 1 displays this analysis. This lack of transparency hampers the visual detection of outliers and influential data points in the analysis, e.g. SNP rs17249754 highlighted by a square symbol, which is illustrated further in Figure 2. In Figure 2 (top), we plot the value of each individual variant’s contribution to Cochran’s *Q* statistic, which is approximately Chi-squared distributed with 1 df under the previously stated assumptions. For these data, *Q* = 67.09 (df = 25), indicating substantial heterogeneity, but the individual contribution of SNP rs17249754 (the eighth variant in our data frame highlighted by a square) is 28.34. It is therefore responsible for the vast majority of excess heterogeneity amongst the 26 ratio estimates. Figure 2 (bottom left and right) shows the Cook’s distance and Studentized residual measures for each variant, which were first used by Corbin *et al*.^13^ to look for influential SNPs in an MR context. Both measures also confirm rs17249754 as *the* major outlier for these data. However, this fact would not be immediately obvious from a visual inspection of the scatter plot alone.

## Methods

### The Radial MR plot

**Figure 3.**
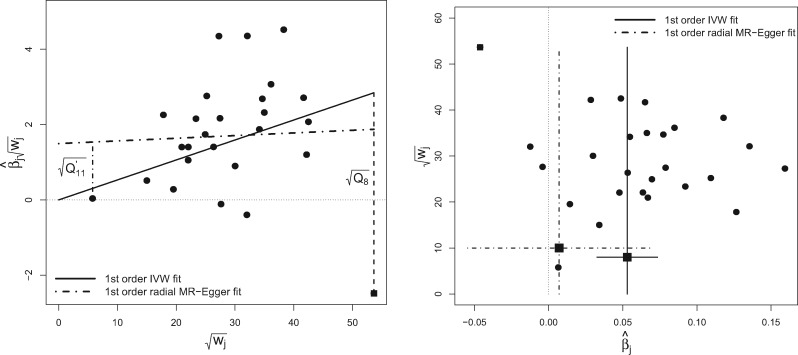
Left: Radial MR plot of the blood-pressure data. IVW and Radial MR-Egger regression slopes calculated using first-order weights are overlaid. The square-root contribution of SNP rs17249754 to Cochran’s *Q* statistic ($\sqrt{Q_{8}}$) is denoted by the vertical dashed line from the IVW slope. The square-root contribution of a separate SNP to Rücker’s $Q^{\prime }$ statistic ($\sqrt{Q_{11}}$) is denoted by the vertical dashed line from the Radial MR-Egger slope. Right: Generalized funnel plot of same data with first-order IVW and Radial MR-Egger regression slopes (and 95% confidence intervals) shown. SNP rs17249754 is shown as a square.

The Galbraith Radial plot^14^^,^^15^ was proposed as a graphical tool to visualize estimates of the same quantity with varying precisions. Specifically, it plots the *Z*-statistics for each estimate (i.e. the point estimate divided by its standard error) on the vertical axis vs the inverse standard error on the horizontal axis. In our notation, the inverse standard error of the *j*th estimate is denoted by $\sqrt{w_{j}}$, where *w* stands for ‘weight’. It has been used extensively in meta-analysis to detect heterogeneity and small-study bias.^12^^,^^16^^,^^17^ We believe that, when translated to the MR setting, it offers a simple solution to the inherent deficiencies of the standard scatter plot. The horizontal axis of the Radial plot is the square root of the actual weight each SNP receives in the IVW analysis. Its vertical-axis scale represents the ratio estimate for each SNP multiplied by the same square-root weight. Since the square-root weight on the horizontal axis is naturally positive, and the vertical axis is a function of this same weight and the ratio estimate (which is coding invariant), the Radial plot removes the need to manually reorient the summary data estimates. Figure 3 (left) shows the blood-pressure data, this time represented on the Radial MR plot. The IVW estimate is again overlaid on top.

The Radial plot still enables the slope joining each data point to the origin to be interpreted as a ratio estimate. A second vertical axis is usually drawn on the right-hand side of the Radial plot as an arc to accentuate this point. We leave this out in this instance in order to focus attention on the new scale of the horizontal and vertical axes only.

An additional helpful property of the Radial plot is that the absolute vertical distance from each data point to the fitted IVW slope is equal to the square root of its contribution to Cochran’s *Q* statistic. From the Radial plot, we can instantly see that SNP rs17249754 is the most influential variant in the IVW analysis, for two reasons:

- it gets the most weight because of its position on the horizontal axis;
- it has the largest contribution to Cochran’s *Q* statistic because it is farthest away from the IVW slope.

The presence of a strong and (potentially) highly pleiotropic instrument is problematic because it calls into question the validity of the InSIDE assumption. Although both the IVW and MR-Egger regression models rely on this assumption, MR-Egger is known to be more sensitive to its violation.^3^

### MR analysis via Radial regression

Although the standard meta-analysis formula can be used to derive the IVW estimate (Box 1), in practice, it is often convenient to obtain the estimate by fitting a linear-regression model. This is a simple command in any software package and allows the user to benefit from the host of summary and diagnostic tools that compliment it. For example, regressing the SNP–outcome associations on the SNP–exposure associations with the intercept constrained to 0 and weighting the regression by the SNP–outcome association standard error will yield the IVW estimate using first-order weights. More generally, we can interpret the IVW estimate calculated using *any* set of user-defined weights as a best-fitting line through the data points on the Radial plot under the constraint that the line goes through the origin. See Box 4 for further details.

**Box 4: Two-sample summary data MR via Radial plot regression**

**Radial IVW regression:** The IVW estimate obtained using any set of weights *w_j_* can be interpreted as the *β* coefficient estimated from the following IVW Radial regression model:

$${\hat{\beta }}_{j}\sqrt{w_{j}}=\beta \sqrt{w_{j}}+\epsilon_{j},\;\epsilon_{j}∼N(0,1).$$

Cochran’s *Q* statistic must then be calculated as

$$Q=\sum_{j=1}^{L}w_{j}{\left({\hat{\beta }}_{j}-{\hat{\beta }}_{IVW}\right)}^{2}.$$

**Radial MR-Egger regression:** As a natural complement to the Radial IVW model above, the following Radial MR-Egger regression model can instead be used to estimate the causal effect:

$${\hat{\beta }}_{j}\sqrt{w_{j}}=\beta_{0E}+\beta_{1E}\sqrt{w_{j}}+\epsilon_{j}.$$

That is, Radial MR-Egger is a regression directly on the Radial plot scale with the intercept parameter left unconstrained. Under a Radial model, the InSIDE assumption is that the pleiotropic effects are independent of the Radial weights.

Rücker’s $Q^{\prime }$ statistic for the Radial MR-Egger model is defined as:

$$Q^{\prime }=\sum_{j=1}^{L}Q_{j}^{\prime }=\sum_{j=1}^{L}w_{j}{\left({\hat{\beta }}_{j}-\frac{{\hat{\beta }}_{0E}}{\sqrt{w_{j}}}-{\hat{\beta }}_{1E}\right)}^{2}.$$

The two main advantages of Radial MR-Egger are (i) it avoids recoding of the genetic data and (ii) it can be interpreted as the best-fitting line through the Radial plot data. This means that the Radial plot residuals are proportional to the square root of their individual contribution to Rücker’s $Q^{\prime }$ statistic above. Fixed-effect and random-effects versions of Radial IVW and Radial MR-Egger regression can be implemented by altering the definition of *w_j_*.

**How does this differ from traditional MR-Egger?** The originally proposed MR-Egger regression model, which implicitly used first-order weights, is equivalent to the following Radial MR-Egger regression model:

$${\hat{\beta }}_{j}\sqrt{w_{j}}=\frac{\beta_{0E}}{\sigma_{Yj}}+\beta_{1E}\sqrt{w_{j}}+\epsilon_{j},$$

where *w_j_* represents first-order weights. That is, $\beta_{0E}$ in the original model is not a true intercept (i.e. a constant); it is the coefficient of the explanatory variable $\sigma_{Yj}^{-1}$, as explained in Ref.^3^ In practice, traditional and Radial MR-Egger will yield qualitatively similar inferences, although the magnitude of their respective intercept parameters will be different. For example, in Table 1, we see that the Radial MR-Egger slope of 0.007 is very similar to the MR-Egger slope of –0.002, but the Radial MR-Egger intercept is 1.5 compared with the MR-Egger intercept of 0.033. Figure 5 shows the Rücker model-selection framework applied to the IVW and Radial MR-Egger regression models.

**Figure 4.**
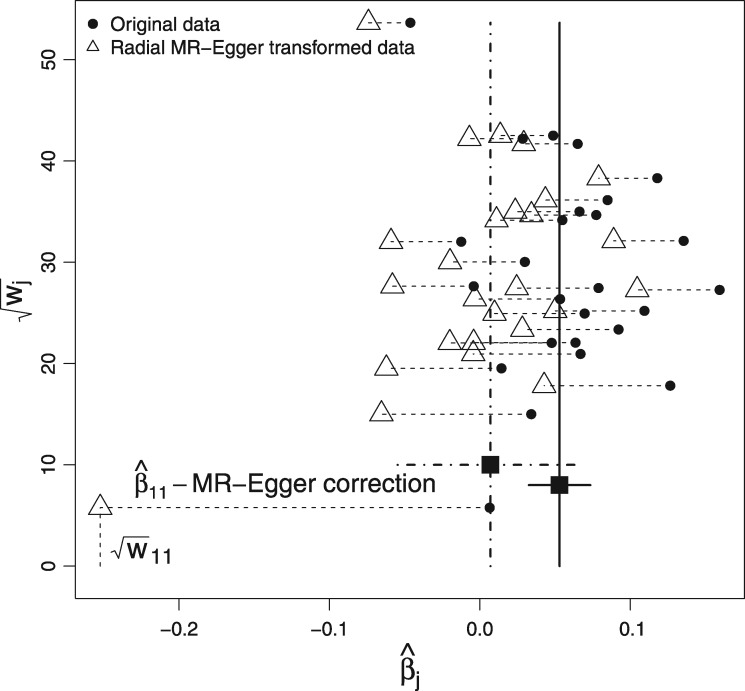
Radial MR-Egger funnel plot. Horizontal dashed lines link the position of data in the standard funnel plot (circles) to their implied position under a Radial MR-Egger analysis (triangles).

**Generalized Radial funnel plots** A generalized Radial funnel plot that naturally complements the Radial plot can be produced by plotting $\sqrt{w_{j}}$ on the vertical axis against ${\hat{\beta }}_{j}$ on the horizontal axis. This plot, however, is most informative for the IVW analysis because the IVW slope lies at the (IVW) centre of the data. An equivalent Radial MR-Egger funnel plot with the same property can be produced by plotting $\sqrt{w_{j}}$ on the vertical axis against the ‘corrected’ ratio estimate

$$\;{\hat{\beta }}_{j}-\frac{{\hat{\beta }}_{0E}}{\sqrt{w_{j}}}$$

on the horizontal axis. Figure 4 shows the Radial funnel plot for our data example.

Just as for the IVW estimate, MR-Egger regression can also be implemented as a linear regression directly on the Radial plot, but with the intercept left unconstrained. We call this *Radial MR-Egger regression*. Radial MR-Egger regression is different from traditional MR-Egger regression, even when first-order weights are used, because the intercept parameter is estimated on a different scale. Estimates obtained from a Radial MR-Egger regression will be consistent for the causal effect as long as the InSIDE assumption is satisfied on this new scale (see Box 4).

Figure 3 (left) shows the Radial MR-Egger regression slope, estimated assuming first-order weights. Just as for the IVW method, the absolute distance from any data point to the Radial MR-Egger slope is equal to the square root of its contribution to the overall heterogeneity after adjustment for pleiotropy—which is measured for MR-Egger by Rücker’s $Q^{\prime }$ statistic. This is illustrated in Figure 3 for a single SNP. Note that the definition of Rücker’s $Q^{\prime }$ is also slightly modified under this analysis (Box 4). The Radial plot can therefore be used to simultaneously assess whether individual variants are outliers with respect to either the IVW or Radial MR-Egger regression models.

### Radial MR-Egger funnel plot

Figure 3 (right) shows the blood-pressure data represented on the funnel plot. It plots the ratio estimate for each variant on the horizontal axis against its square-root precision (or weight) on the vertical axis. In this instance, first-order weights were used to scale the vertical axis and to calculate the IVW and Radial MR-Egger regression slope estimates, which are overlaid on top. Under first-order weighting, Figure 3 (right) is equivalent to the funnel plot first used by Bowden *et al.*^11^ to visualize MR analyses and to look for asymmetry as a sign of pleiotropy. However, we label the vertical axis generically to stress that a Radial funnel plot can be produced, and will naturally compliment its corresponding Radial plot, when *any* given set of weights is used.

Although it is possible to interpret the Radial plot simultaneously for IVW and Radial MR-Egger regression, the funnel plot in Figure 3 (right) is predominately informative about the IVW analysis. Specifically, the IVW estimate intuitively lies in the ‘centre of mass’ of the data when the mass of each ratio estimate is equated with its weight. This is explained in detail by Bowden and Jackson.^18^ In order to produce a funnel plot with this same property for Radial MR-Egger, we must apply a transform to the ratio estimate of each data point in the funnel plot, by subtracting the Radial MR-Egger intercept estimate divided by the ratio estimates’ square-root weight^18^ (see Box 4). This is shown by the horizontal dashed lines in Figure 4. Because it is inversely proportional to the square-root weight, the correction will be larger for imprecise ratio estimates and smaller for precise estimates. The correction factor for the least precise (11th) ratio estimate, ${\hat{\beta }}_{11}$, is explicitly labelled. We can relate and cross-reference this to the corresponding Radial plot in Figure 3 (left), where the 11th ratio estimate is also labelled. It is not an outlier in the IVW analysis because of its proximity to the IVW slope, but its distance from the Radial MR-Egger slope is far greater.

## Results

Table 1 shows the results of our re-analysis of the blood-pressure data using IVW and MR-Egger regression, first with all 26 SNPs and then with SNP rs17249754 removed. For comparison, we show results for both the standard and Radial implementations of MR-Egger regression. All analyses were carried out using first-order weights and assuming a multiplicative random-effects model if any residual heterogeneity was detected.

**Table 1.** IVW and MR-Egger regression analyses of the SBP data with all SNPs and with SNP rs17249754 removed. Multiplicative random-effects models were fitted in all cases whenever over-dispersion was detected

Model/parameter	Est.	S.E.	*t*-value	*p*-value	Heterogeneity statistic
Complete data					
IVW					
*β_IV W_*	0.0531	0.0104	5.08	3.01 × 10^– 5^	*Q* = 67.09 (*p* = 1 × 10^–5^)
MR-Egger					
$\beta_{0E}$	0.033	0.018	1.86	0.075	–
$\beta_{1E}$	–0.002	0.031	–0.078	0.939	$Q^{\prime }$ = 58.60 (*p* = 1 × 10^–4^)
Radial MR-Egger					
$\beta_{0E}$	1.495	0.967	1.54	0.136	–
$\beta_{1E}$	0.007	0.0315	0.225	0.824	$Q^{\prime }$ = 61.05 (*p* = 4.5 × 10^–5^)
SNP rs17249754 removed					
IVW					
*β_IV W_*	0.066	0.008	8.08	2.63 × 10^– 8^	*Q* = 35.00 (*p* = 0.068)
MR-Egger					
$\beta_{0E}$	0.010	0.015	0.670	0.509	–
$\beta_{1E}$	0.049	0.027	1.760	0.092	$Q^{\prime }$ = 34.33 (*p* = 0.061)
Radial MR-Egger					
$\beta_{0E}$	0.059	0.826	0.071	0.944	–
$\beta_{1E}$	0.064	0.028	2.294	0.031	$Q^{\prime }$ = 34.99 (*p* = 0.052)

The IVW estimate for the causal effect of a 1-mmHg increase in SBP on the log-odds ratio of CHD is 0.053. Large heterogeneity is present amongst the 26 ratio estimates, as identified by Cochran’s *Q*, which is sufficiently extreme (*p* = 1 × 10^–5^) to opt for a random-effect IVW model instead. Standard and Radial MR-Egger regression yield qualitatively similar results and suggest a causal effect close to 0. Both models represent a better fit to the data at well below the conventional 5% threshold, since, in each case, $Q-Q^{\prime }$ is much larger than 3.84 (the 95th percentile of a Chi-squared distribution on 1 df). Since a large amount of residual heterogeneity was still present around both the standard and Radial MR-Egger fits (as detected by $Q^{\prime }$), their standard errors were also inflated to allow for over-dispersion.

When the three analysis methods are repeated this time with variant rs17249754 removed, IVW and MR-Egger causal estimates are virtually identical, especially with those of Radial MR-Egger. Cochran’s *Q* and Rücker’s $Q^{\prime }$ statistic only reveal a small amount of residual heterogeneity and examination of $Q-Q^{\prime }$ reveals neither standard nor Radial MR-Egger represents a better fit to the data than the IVW model. Therefore, the data do not support a move away from the standard IVW analysis without SNP rs17249754.

### A leave-one-out sensitivity analysis

**Figure 5.**
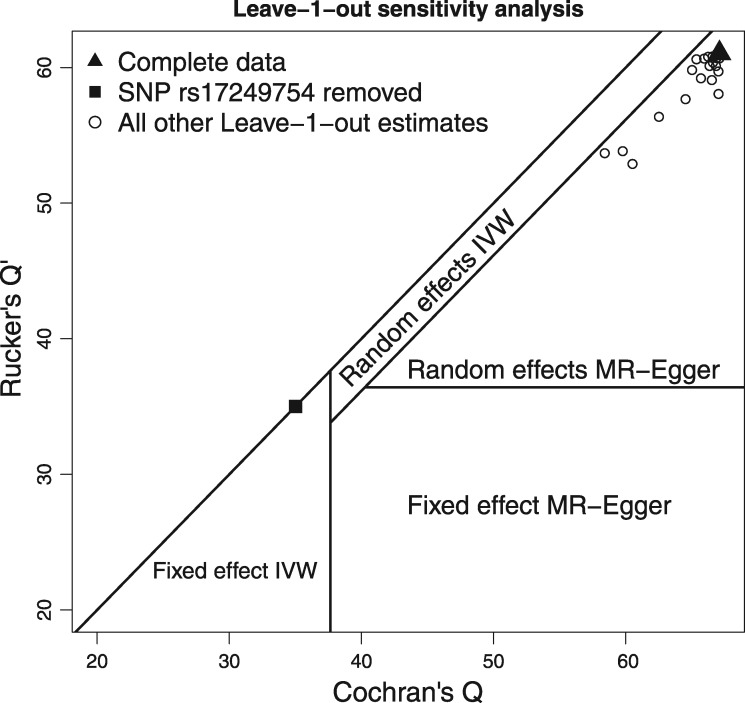
Leave-one-out sensitivity analysis of the data, showing the values of *Q* and $Q^{\prime }$ when each variant is left out of the analysis in turn. Points are overlaid on the Rücker decision space that governs which of four model choices should be favoured. It assumes a significance threshold of *δ* = 0.05 to affect the model selection.

Rather than using the Rücker framework for formal model-selection purposes (Box 3), we instead demonstrate its utility in providing a useful, but informal, backdrop to assess the influence of each individual variant on the analysis under the IVW and MR-Egger frameworks. Figure 5 shows the values of Cochran’s *Q* (calculated with respect to the IVW fit) against Rücker’s $Q^{\prime }$ (calculated with respect to the Radial MR-Egger fit) for 26 analyses where each SNP is left out in turn. These points are overlaid on top of the Rücker decision space assuming a threshold of *δ* = 0.05 for declaring heterogeneity using *Q* and $Q^{\prime }$. In the main analysis reported in Table 1, random-effects models were fitted if *any* heterogeneity at all was detected, which is equivalent to setting *δ* = 0.5. The nested nature of the Radial IVW and MR-Egger models guarantees that all points in Figure 5 lie below the diagonal line *Q* =$Q^{\prime }$.

When all the data are analysed together (triangular point in Figure 5), sufficient heterogeneity and bias are detected to mean that a random-effects Radial MR-Egger regression model is best supported by the data. It infers the presence of large directional pleiotropy and no causal effect between SBP and CHD risk. This is not materially changed when every variant *except* SNP rs17249754 is left out of the analysis in turn (circular points in Figure 5). However, when SNP rs17249754 is removed from the data (square point in Figure 5), there is no evidence of heterogeneity or bias due to directional pleiotropy and the data provide no reason to move away from a standard IVW analysis.

### The Radial plot function

**Figure 6.**
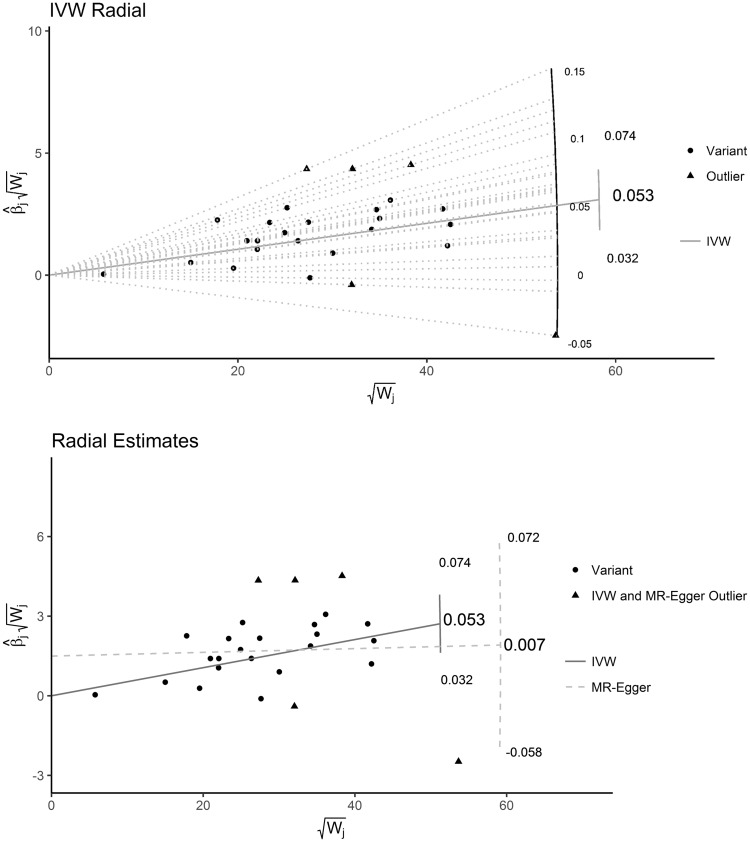
Radial plots of the blood-pressure data produced using the RadialMR package. Top: Only the IVW estimate shown, Radial lines joining each data point back to the origin. Bottom: Radial MR-Egger and IVW model fits shown.

We have written an R package RadialMR to produce Radial plots and to perform Radial regression. Two of the many possible plot options are illustrated for the blood-pressure data in Figure 6. Figure 6 (top) shows the Radial plot of the IVW analysis alone, which includes a Radial curve to highlight the ratio estimate for each genetic variant, as well as the overall IVW estimate. Data points with large contributions to Cochran’s *Q* statistic are shown in orange. The significance level for identifying these outliers can be set by the user; here we chose the value 0.01. Figure 6 (bottom) shows the Radial plot on a tighter scale, with both IVW and Radial MR-Egger regression implemented. Outliers for either method (and both methods) are shown. A table of the exact *Q* and $Q^{\prime }$ contributions for each variant is given as an output for the researcher to conduct a more detailed analysis.

Radial plots are produced by many existing R packages such as *metafor, numOSL* and *Luminescence*. Care will need to be taken, however, to input data from an MR analysis appropriately into these generic platforms. For this reason, we will also continue to develop our own RadialMR package to keep pace with the latest developments in the field of MR. It is currently available to download at https://github.com/WSpiller/RadialMR/.

### Conclusion

It has long been appreciated in the general meta-analysis context that the Radial plot has many desirable characteristics over the traditional scatter plot, especially in the detection of outlying studies and small-study bias. Given its intimate connection with meta-analysis, we propose that the Radial plot should also be given a more central role in two-sample summary data MR studies.

The Radial plot, and its corresponding funnel plots, improve the visual interpretation of data used within an MR analysis because it provides the most transparent representation from an information-content perspective. Its implications stem beyond the purely aesthetic for MR-Egger regression, however. Radial MR-Egger is an attractive modification and generalization of the original approach that naturally flows from the use of this plot. On top of removing the need to recode the genetic data and facilitating a more straightforward detection of outliers, the Radial formulation also makes it much more transparent that it is attempting to detect any systematic trend in ratio estimates according to the weight they receive in the analysis. Another advantage is that it only requires the ratio estimates and their standard errors. This makes it applicable even when data on individual SNP–exposure and SNP–outcome associations (and their standard errors) are not available.

When first-order weights are used, Radial MR-Egger and traditional MR-Egger will generally yield similar causal estimates, but the magnitude of the intercept will be different. An undoubted strength of the Radial approach lies in the fact that it can be seamlessly applied when any set of weights is used. In recent work, Bowden *et al.*^4^ have shown that first-order weights can inflate the type I error rate of Cochran’s *Q* statistics for detecting heterogeneity, whenever the SNPs utilized are weak instruments or there is a large causal effect. Conversely, second-order weights can dramatically reduce the power of detecting heterogeneity when it is truly present. They propose iterative and exact weights that depend on the causal estimate to improve the performance of the IVW estimate and Cochran’s *Q* statistic. These weights (and indeed any weights) can be immediately incorporated into a Radial plot and the IVW approach. In future work, we will extend this approach for Radial MR-Egger also. Further investigation into the properties of Radial MR-Egger in a variety of circumstances is required, but the features that distinguish it from the standard approach appear attractive, and it has the potential to become *the* standard implementation.

When conducting a two-sample summary data MR analysis with a binary outcome, natural correlations will exist between causal-effect estimates (e.g. log-odds ratios) and their precisions, which could easily contribute to heterogeneity and hence be misconstrued as pleiotropy. In related work on the meta regression of separate trial results measuring a binary outcome, Harbord *et al.*^19^ show that regressing the ratio of the score and square-root information statistics against the square-root information (in a close analogy to the Radial plot) is better at mitigating this effect than simply working directly with the log-odds ratio and its standard error. As further work, we plan to extend the approach of Harbord to the MR context for Radial MR-Egger regression with binary outcomes. Similar approaches based on score and information statistics may also prove useful for MR analyses of time-to-event outcomes.

We illustrated a leave-one-out analysis using the Rücker model-selection framework as a backdrop when conducting an MR study, to understand how model choice is affected by the exclusion of individual variants. However, we stress some caution in following this approach to the extreme using a purely statistical criterion, e.g. in adopting a strategy of removing all outliers until little or no heterogeneity remains. Procedures such as this have been proposed when meta-analysing separate study results,^20^ but have been criticized for being too data-driven, likely to throw out larger studies than smaller studies and offering little explanation as to the underlying cause of heterogeneity.^21^ A much stronger criterion for exclusion of a particular SNP would be to first detect it as a statistical outlier and then confirm the SNP’s association with a separate phenotype that represents a pleiotropic pathway to the outcome.

The Rücker model-selection framework we present explores how the choice of IVW or MR-Egger model is affected by the summary data from each SNP, but it cannot tell the user about the probability that each model is true. Thompson *et al.*^22^ have proposed a formal Bayesian model averaging framework that achieves this aim and produces posterior causal-effect estimates accounting for model uncertainty. Hemani *et al.*^23^ have also recently proposed a machine learning framework for choosing between a much larger group of modelling choices. Both ideas nicely compliment and extend the basic approach outlined here.

**Conflict of interest:** None declared.
